# Development of a Multimatrix UHPLC-MS/MS Method for the Determination of Paracetamol and Its Metabolites in Animal Tissues

**DOI:** 10.3390/molecules26072046

**Published:** 2021-04-02

**Authors:** Konrad Pietruk, Małgorzata Gbylik-Sikorska, Beata Łebkowska-Wieruszewska, Anna Gajda, Mario Giorgi, Irene Sartini, Piotr Jedziniak

**Affiliations:** 1 Department of Pharmacology and Toxicology, National Veterinary Research Institute, 24-100 Pulawy, Poland; malgorzata.gbylik@piwet.pulawy.pl (M.G.-S.); anna.gajda@piwet.pulawy.pl (A.G.); piotr.jedziniak@piwet.pulawy.pl (P.J.); 2 Department of Pharmacology, Toxicology and Environmental Protection, University of Life Sciences, 20-950 Lublin, Poland; lebkowska.wieruszewska@up.lublin.pl; 3 Department of Veterinary Sciences, University of Pisa, 56126 Pisa, Italy; mario.giorgi@unipi.it; 4 Department of Veterinary Medicine, PhD School, University of Sassari, 07100 Sassari, Italy; i.sartini@studenti.uniss.it

**Keywords:** paracetamol, metabolites, muscle, heart, lungs, liver, kidneys, UHPLC-MS/MS, residues

## Abstract

Paracetamol/acetaminophen (APAP) is one of the most popular pharmacologically active substances used as an analgesic and antipyretic agent. The metabolism of this drug occurs in the liver and leads to the formation of two main metabolites—glucuronic acid and sulfate derivate. Despite the wide use of paracetamol in veterinary medicine, a handful of analytical methods were published for the determination of paracetamol residues in animal tissues. In this paper, a multimatrix method has been developed for the determination of paracetamol and two metabolites—paracetamol sulfate (PS) and p-Acetamidophenyl β-D-glucuronide (PG). A validation procedure was conducted to verify method reliability and fit purpose as a tool for analyzing acetaminophen and metabolites in muscle, liver, lung, and kidney samples from different species of animals. Established validation parameters were in agreement with acceptable criteria laid by the European legislation. The initial significant matrix effect was successfully reduced by implementing an internal standard—4-Acetamidophenyl β-D-glucuronide-d3 (PG-d3, IS). The usefulness of the developed method was verified by analyzing samples from an experiment in which paracetamol was administrated to geese.

## 1. Introduction

Paracetamol/acetaminophen (N-acetyl-para-aminophenol; APAP) is an analgesic and antipyretic agent [1,2,3,4,5]. Paracetamol is one of the major metabolites of acetanilide and phenacetin [1]. Although paracetamol was synthesized in 1878 [2], clinically used in 1887 [6], and first marketed to consumers in the 1950s in the USA [7], its mechanism of action is still not fully understood [1,2,4]. It provides analgesic and antipyretic effects similar to nonsteroidal anti-inflammatory drugs (NSAIDs) but does not produce any anti-inflammatory effects [1,2]. Paracetamol possesses an analgesic mechanism of action that is a very complex process, in which both peripheral and central antinociceptive processes must be considered, as well as the redox mechanism [2].

It is widely used in human medicine over the counter (OTC) painkillers because of its effective action and relative safety (at therapeutic doses) [1,4,8,9]. It is also one of the most commonly used antipyretics and analgesics in pediatrics and the only one recommended for use in newborns [6,10]. Paracetamol can also be used to treat livestock such as pigs, cattle, or poultry [11]. It is one veterinary medicinal product (VMP) authorized in the medicated pre-mixes in Denmark [12]. Paracetamol is also classified as a pharmacologically acceptable active substance for use in pigs, but no maximum residue limit (MRL) has been established for this compound so far [13].

Metabolism of paracetamol occurs in the liver through various pathways that lead to the formation of mainly inactive metabolites, glucuronic acid and sulfate conjugates, both accounting for about 80–90% of doses of paracetamol (non-oxidative metabolism) [2,3,14,15]. Oxidative metabolism of paracetamol is based on the production of the reactive *N*-acetyl-p-benzoquinone imine (NAPQI), a toxic metabolite. It is catalyzed by cytochrome P450 but rapidly metabolized and detoxified to the glutathione conjugate. This process can be disrupted by increasing the dose of paracetamol, which may result in hepatotoxicity and nephrotoxicity [3,4,5,8,11,16,17,18].

In the literature, information on the metabolism of paracetamol in plasma [7,19,20,21,22] or urine [22] in humans and animals is available. However, there is no information on the presence and concentration of its main metabolites in animal tissues. There are also conducted studies on the pharmacokinetics of paracetamol or/and its metabolites in humans [20,21,22], laboratory animals such as mice [23] and rats [24], in livestock [25,26] and companion animals [27]. However, there is a complete lack of knowledge on the depletion of paracetamol and its metabolites in food-producing animal tissues (poultry, cattle) other than pigs [11].

Conducting such studies requires the development of appropriate analytical methods, which make it possible to determine these substances in different tissues. There are many papers in the literature describing analytical methods for the determination of paracetamol in plasma or urine [7,19,20,21], paracetamol, and its metabolites in human and animal plasma and urine [22,23,24]. Work by Zhang describes a multimatrix method for the determination of paracetamol and metabolites in mouse plasma, liver, and kidney samples [28]. The scope of this method includes NAPQI, acetaminophen-glutathione, and acetaminophen-glucuronide. Determination of paracetamol and metabolites in the unconventional matrix such as a dried blood spot was also reported [29]. However, to the best of our knowledge, there are only a few papers on the determination of paracetamol in animal meat [30] or muscle [31] and absolutely no papers describing the determination of paracetamol and its metabolites in different tissues and species from food-producing animals.

The purpose of this study was to develop the multimatrix UHPLC-MS/MS method for the determination of paracetamol (APAP) and its metabolites—paracetamol glucuronide (PG) and paracetamol sulfate (PS). The main advantage of this method would be its capability for analyzing samples, both from different animal species (swine, poultry, and cattle) and a wide range of matrices (muscle, lungs, liver, and kidneys). This developed method was validated according to Commission Implementing Regulation (EU): SANTE/11188/2018 CIS draft document [32]. Linearity, selectivity, specificity, precision (repeatability and within-laboratory reproducibility), decision limit (CCα), detection capability (CCβ), recovery, and matrix effect were evaluated. This method has been successfully applied to the determination of paracetamol and its metabolites in real samples.

## 2. Results and Discussion

### 2.1. Optimization of LC-MS/MS Conditions

We started developing an analytical method optimizing the MS/MS condition by injecting a standards solution of paracetamol and its metabolites. The most abundant signal for paracetamol was achieved in positive ionization mode, but for paracetamol metabolites, negative ionization mode was found to be more sensitive. In previously published papers, the positive mode for paracetamol [19,20,21] and paracetamol metabolites [22,23,28] was used. On the other hand, analyzing paracetamol and metabolites in dried blood spots was conducted using negative ionization [29]. Only a single paper described an approach using positive ionization for paracetamol and negative ionization for metabolites [24].

Deciding on this approach for 4-Acetamidophenyl β-D-glucuronide-d3 sodium salt (PG-d3, IS), positive and negative transitions were monitored. Detailed mass spectrometric conditions are shown in Table 1.

The chromatographic conditions were optimized to achieve a suitable peak shape and sensitivity. For this purpose, various mobile phase combinations and chromatographic columns were investigated. The optimal results were achieved using a solution of formic acid with acidic acetonitrile and Agilent Zorbax Eclipse Plus C18 column. In previously published papers, depending on matrix, combinations of acetic acid with methanol [19] or formic acid with acetonitrile [21,23,24] were used. Another study used ammonium acetate containing acetic acid solution with acetonitrile as a mobile phase [20]. Chromatographic separation in all previously published methods was performed using C18 columns [19,20,21,22,23,24].

### 2.2. Optimization of Sample Preparation

The scope of our method covered samples such as liver, muscle, lungs, and kidneys from different animal species. The main goal for sample preparation of our method was quite ambitious and challenging: one extraction and clean-up scheme for all different matrices.

**Figure 1:**
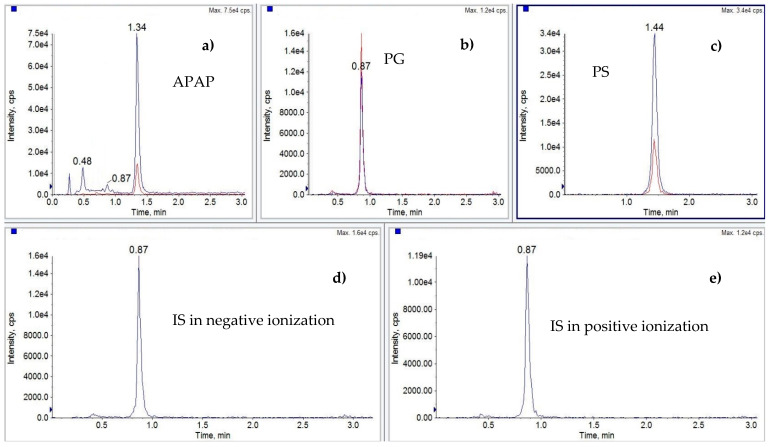
UHPLC-MS/MS XICs, control muscle sample from geese spiked with: (**a**) paracetamol (APAP), (**b**) p-Acetamidophenyl β-D-glucuronide sodium salt (PG), (**c**) Paracetamol sulfate potasium (PS), (**d**) and (**e**) 4-Acetamidophenyl β-D-glucuronide-d3 sodium salt (PG-d3) IS in negative and positive ionization, respectively. Paracetamol and metabolites were spiked at 50 µg/kg and internal standard at 25 µg/kg.

**Figure 2:**
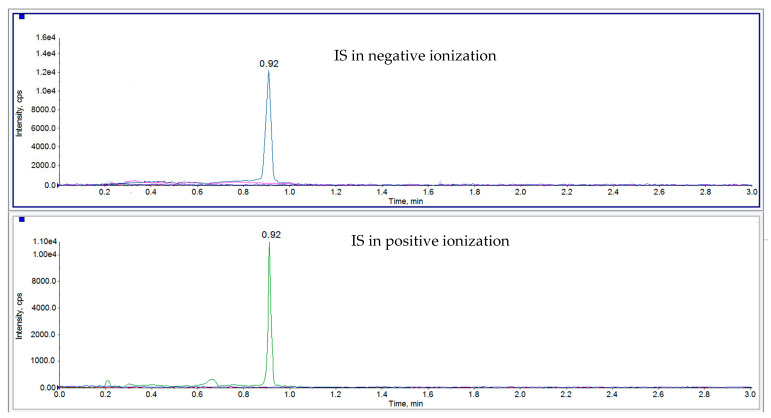
UHPLC-MS/MS XICs, blank muscle sample from geese spiked with 4-Acetamidophenyl β-D-glucuronide-d3 sodium salt (PG-d3) IS at 25 µg/kg in negative and positive ionization, respectively.

The most satisfactory extraction recovery for all analytes was acquired by using a two-step extraction procedure. Firstly, 4 mL of acetonitrile was applied, followed by 4 mL of methanol containing 0.1% of formic acid. Due to the complexity of matrices in the scope of the method, a clean-up step was required. During optimization C18, SiOH, and polymer SPE cartridges were evaluated. The most promising results for all matrices were obtained on SiOH SPE cartridges, which were included in the final version of the method. As an example, a chromatogram of muscle samples spiked at 50 µg/kg with all three analytes and blank sample are presented in Figure 1 and Figure 2, respectively.

A few papers were published regarding the determination of paracetamol in meat or muscles. Jian et al. used EDTA-McIlvaine’s buffer as an extraction solvent to determine paracetamol and chloramphenicol in meat samples followed by a cleanup step using polyaniline [27]. The method developed by Hu et al. had a much more broad scope of analytes. Beyond paracetamol, their method was capable of determining residues of 29 other (nonsteroidal anti-inflammatory) drugs in swine muscle. The extraction protocol consisted of acetonitrile with the addition of phosphoric acid with a comprehensive clean-up procedure including hexane saturated with acetonitrile and SPE cartridges [28].

### 2.3. Method Validation

Criteria for validation parameters for analytical methods used in National Monitor Control Plans in the European Union are described in Commission Decision 657 from 2002 [33]. Soon the above-mentioned legislation will be repealed; therefore, we decided to perform a validation experiment using a draft of guidelines described in the Commission Implementing Regulation SANTE/11188/2018 document [29], which would be included in the document replacing CD/657/2002.

Linearity of matrix-matched calibration curves was acceptable for all analytes in all matrices for the lower range (50–500 µg/kg) with values for R^2^ above 0.98. In the case of a higher range of calibration curves, the initial range (1000–10000 µg/kg) was confirmed only for lung and kidneys for all analytes. For muscle and liver to linearity was confirmed for range from 1000 to 5000 µg/kg.

The limit of detection (LOD) based on signal-to-noise ratio was calculated to be equal to 10 µg/kg for all analytes in all matrices. The limit of quantification (LOQ) of the method was set as the lowest point of the calibration curve (50 µg/kg) for all matrices with an acceptable coefficient of variation values. Based on results acquired for samples fortified at 50 µg/kg for all analytes, it might be possible to obtain even lower LOQ if needed.

The study’s selectivity revealed no interference peaks in analyzed samples. Repeatability and within-laboratory results were in line with criteria described in the draft of the Commission Implementing Regulation document. Repeatability and within-laboratory reproducibility measured as CV (%) in all cases were below 20% with the exception for PG in muscle samples—CV = 21.0%. Calculated values of CCα and CCβ were similar across all analytes and matrices. Slightly higher values for PS and PG were found in muscle samples, which are a result of higher values of CV in this particular case. Recovery is also in a satisfactory range for all analytes. It should be emphasized that validation was performed using a mixture of samples from different animal species for each matrix; still, results are in agreement with criteria in SANTE/11188/2018. Detailed results of the validation experiment are shown in Table 2. The result of stability studies of the individual stock standard solution showed that all analytes were stable at least for six months at −18 °C. A mixture of working standard solution and IS solution stored at the same temperature also were stable for six months. There were no differences in the stability of the individual substances under the storage conditions tested. The differences between peak area of freshly-prepared and seven-months-stored solutions ranged from 25–40% for all compounds. The developed method is rugged because none of the factors tested affected the precision (reproducibility) of the method.

**Figure 3:**
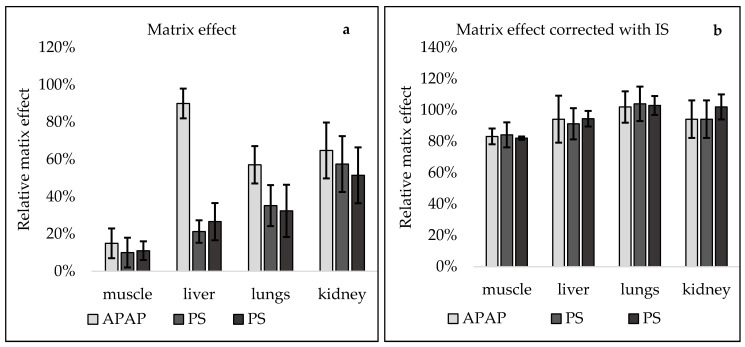
Matrix effect (mean ± SD, *n* = 20) in muscle, liver, lung, and kidneys samples: (**a**) without internal correction and (**b**) with internal correction.

Finally, the matrix effect was evaluated based on the post-extraction addition technique. The most prominent matrix effect was observed in muscle samples for all three analytes. Ion suppression caused more than 85% of signal reduction. In liver and lungs for PS and PG, significant ion suppression (70–80%) was also observed. For paracetamol, those values were much lower; in the liver, only 10% of the ion suppression effect was noted, but in the lungs, almost 40% of the matrix effect caused by ion suppression was detected. The lowest impact of the matrix effect was observed in the case of kidney samples. The reduction of the signal was mostly on the same level for paracetamol and metabolites and was in the range of 40–50%. Such high values of the matrix effect are not surprising; other authors also reported significant ion suppression in their developed methods [34,35,36,37]. Introducing internal standards is considered to be one of the easiest and most effective solutions to reduce the matrix effect in analytical methods. In the present study, a major improvement was observed when correction with internal standards was performed. The most prominent improvement was noted for muscle samples, in which the matrix effect was the biggest issue. For all the analytes, ion suppression was reduced to less than 20%; for other matrices improvement was also detected. Graphical presentation of matrix effect with and without IS correction is presented in Figure 3.

### 2.4. Application to Real Samples

The applicability of the developed method was checked by analyzing samples obtained from the animal experiment in which the pharmacokinetics and depletion curve of APAP and its metabolites were evaluated. Acetaminophen was orally administrated to geese (single dose 10 mg/kg). Detailed results of pharmacokinetics and depletion profile will be presented in the following paper. Several samples (*n* = 8) (muscle, liver, kidneys, and lungs) were analyzed 10 h after drug administration. Initial validation was done on samples containing a mixture of animal species. Therefore, to confirm methods’ capability with samples containing only goose material, repeatability and reproducibility were verified.

The highest concentration of APAP was found in liver samples—918 ± 73 µg/kg. In the lungs, the highest amount for PS and PG was detected—966 ± 86 and 1120 ± 97 µg/kg, respectively. Concentrations in muscle samples were on a similar level for all analytes ranging from 174 ± 24 µg/kg for APAP to 106 ± 21 µg/kg for PG. In the case of kidney samples, only PG was above the LOQ of the method. Obtained results are summarized in Table 3.

## 3. Materials and Methods

### 3.1. Chemicals and Reagents

The paracetamol (APAP), paracetamol sulfate potassium (PS), p-Acetamidophenyl β-D-glucuronide sodium salt (PG), and 4-Acetamidophenyl β-D-glucuronide-d3 sodium salt (PG-d3) were obtained from Sigma-Aldrich (St. Louis, MO, USA), acetonitrile, methanol both LC-MS grade and SiOH 500 mg SPE cartridges were purchased from Avantor Performance Materials Poland (Gliwice, Poland). Formic acid (99% for LC-MS) was acquired from VWR Chemicals (Randor, USA). Ultrapure water was obtained using a Milli-Q purification system (Millipore, France).

### 3.2. Preparation of the Standard Stock Solution and Working Solutions

Individual standard stock solutions were prepared by dissolving 10.00 mg of each substance in 10 mL of acetonitrile. Afterward, suitable dilutions in acetonitrile were prepared from stock solutions to obtain a mixture of working solutions used in sample spiking. All solutions were stored at −18 °C for six months.

### 3.3. Sample Preparation

Two grams of control (blank) muscle, liver, lung, or kidney sample were weighted into a 50 mL polypropylene centrifuge tube, spiked with 10 µL of internal standard working solution (5 µg/mL), and mixed using vortex (1 min). The sample was left for 10 min to ensure standards dispersal. Then, 4 mL of acetonitrile was added to the analytes sample and vortexed (1 min); afterward 4 mL of 0.1% formic acid in methanol was added and mixed on vortex (1 min). Next, the sample was centrifuged (4845× *g*) for 10 min at 20 °C. Afterward, 6 mL of the supernatant was passed through a 500 mg SiOH SPE cartridge (used as a filter) pre-conditioned with 2 mL of methanol. Filtrated extract was collected in a glass tube and evaporated to dryness using a nitrogen stream at 45 °C. Residues were dissolved in 600 µL of 0.1% formic acid, filtered by 0.22 µm PVDF membrane syringe filters into vials.

### 3.4. LC-MS-MS Analysis

The LC-MS/MS analysis was performed using UHPLC Shimadzu Nexera X2 (Shimadzu, Japan) system connected to the SCIEX 4500 triple quadrupole mass spectrometer (Sciex, USA). Separation of the analytes was performed using Agilent Zorbax RRHD (50 × 2.1 mm, 1.8 µm) column (Agilent, USA) coupled with a guard column. The mobile phase composition was 0.1% formic acid (A) and 0.1% formic acid in acetonitrile (B). Gradient elution was performed with the following program: 0–5 min 95% A, 5–6.3 min 15% A and finally from 6.31 to 8 min back to 95% A. The oven temperature was set to 45 ° Celsius, the flow rate was equal to 0.6 mL/min and the injection volume was set to 10 µl. Detection was conducted in positive and negative electrospray ionization mode (ESI). Two transitions were monitored for paracetamol and metabolites and one transition for IS. Data collection was performed using the analyst 1.6.2 software.

### 3.5. Method Validation

The method was validated according to criteria laid down in the Commission Implementing Regulation SANTE/11188/2018 draft document. The following parameters were established: linearity, selectivity, precision (repeatability and within-laboratory reproducibility), decision limit (CCα), detection capability (CCβ), recovery, matrix effect, and ruggedness. The validation process was carried out on blank samples for each matrix, prepared as a mixture of samples from different species of animals in the following composition: goose 30%, pig 20%, cattle 20%, chicken 15%, and turkey 15%.

Linearity was evaluated by matrix-matched calibration curves. Two calibration curves were prepared: lower range −50, 100, 250, 500 µg/kg and higher range of concentration −1000, 2500, 5000, 10,000 µg/kg. The limit of detection (LOD) was calculated as the signal-to-noise ratio (S/N) ≈ 3. The limit of quantification (LOQ) of the method was set as the lowest point of the calibration curve (50 µg/kg) for which coefficient of variation (CV) was acceptable as described in the Guidance Document on the Estimation of LOD and LOQ for Measurements in the Field of Contaminants in Feed and Food [38].

Linearity was evaluated by calculating the correlation coefficient (R^2^)—plotting area ratios of each analyte versus concentration. Selectivity was established by analyzing 20 blank samples of all matrixes spiked with a standard mixture of nonsteroidal anti-inflammatory drugs. Repeatability was evaluated by spiking six blank samples with a mixture of paracetamol and metabolites on three different levels—50, 75, and 150 µg/kg. Standard deviation and coefficient of variation were calculated for each level. The within-laboratory reproducibility was established by analyzing two additional series in reproducibility conditions within different days and by different analytical personnel. Decision limit (CCα) and detection capability (CCβ) were calculated by using data acquired during the within-laboratory reproducibility study. Recovery was calculated by dividing mean concentrations obtained in reproducibility studies by a particular fortification level. The matrix effect was verified by using the post-extraction addition method [39]. Blank samples were spiked with the standard solution at a concentration of 50 µg/kg at the end of the sample preparation protocol and injected into LC-MS/MS system. The matrix effect was evaluated by analyzing 20 blank samples spiked post-extraction and comparing areas with a standard working solution. The influence of the addition of internal standards on the matrix effect was also checked. The stability of APAP, PS and PG stock standard solution (1000 µg/mL), a mixture of a working standard solution (5 µg/mL), and a mixture of a working internal standard solution (5 µg/mL) were determined at the following periods: 1, 2, 3 weeks, 1, 3, 6 and 8 months. All standard solutions were stored at −18 °C. Ruggedness of the developed method was evaluated by implementing minor changes in sample preparation protocol. The influence of the following parameters on the results was tested: volume of solvents used for the extraction (3.5 mL vs 4.5 mL), different brands of SiOH SPE cartridge provider, the temperature of evaporation (40 °C vs 50 °C), different assay temperatures (19 °C and 21°C), two different analysts and two different days.

## 4. Conclusions

An analytical method for the determination of paracetamol and two metabolites (paracetamol sulfate and p-Acetamidophenyl β-D-glucuronide) in animal tissues has been developed and validated. The reliability of the method was evaluated during the validation experiment. All validation parameters were within the criteria described in the draft of the Commission Implementing Regulation document. During the matrix effect study, relatively high ion suppression was observed, which can be successfully overcome by implementing an internal standard. Applicability of the developed method was evaluated by analyzing samples containing analytes in the scope of the method after administration to animals.

## Figures and Tables

**Table 1:** Summary of the MRM monitored for analytes and MS/MS parameters.

Analyte	Ionization	Parent ion [*m/z*]	Daughter ion(s) [*m/z*] Quantitative/Qualitative	Retention Time (min)	DP [V]	CE [eV]
Paracetamol (APAP)	positive	152.0	110.1/65.1	1.34	45.0	23.0/45.0
Paracetamol sulfate potasium (PS)	negative	230.0	150.0/107.0	1.44	−75.0	−28.0/−46.0
p-Acetamidophenyl β-D-glucuronide sodium salt (PG)	negative	326.0	150.0/107.0	0.87	−50.0	−61.0/−38.0
4-Acetamidophenyl β-D-glucuronide-d3 sodium salt (PG-d3) IS	negative positive	351.0 353.0	150.0 177.0	0.87	-130.0 45.0	−40.0 23.0

**Table 2:** Validation results.

Muscle						
Analyte	Repeatability*, (CV,%)	Within-lab Reproducibility*, (CV,%)	LOQ (µg/kg)	Recovery* (%)	CCα (%)	CCβ (%)
APAP	8.3 ± 2.9	10.8 ± 1.5	50.0	101.4 ± 6.6	63.3	73.1
PS	11,0 ± 1.4	14.0 ± 5.0	50.0	98.2 ± 4.4	76.9	86.7
PG	17.9 ± 4.3	21.0 ± 5.0	50.0	98.2 ± 4.4	76.9	86.7
**Liver**						
APAP	10.8 ± 4.9	11.2 ± 4.5	50.0	110.3 ± 4.6	69.3	79.5
PS	9.5 ± 4.2	12.3 ± 6.3	50.0	114.0 ± 3.4	66.1	76.3
PG	11.5 ± 5.2	15.9 ± 6.3	50.0	108.0 ± 3.4	69.1	79.3
**Lungs**						
APAP	9.8 ± 2.1	10.2 ± 2.5	50.0	110.6 ± 4.6	68.2	78.4
PS	5.4 ± 1.2	7.3 ± 2.1	50.0	109.0 ± 3.4	64.2	74.4
PG	15.5 ± 3.2	18.9 ± 4.1	50.0	91.0 ± 3.4	69.1	79.3
**Kidneys**						
APAP	7.3 ± 2.1	9.7 ± 2.5	50.0	97.6 ± 4.6	69.4	78.9
PS	8.2 ± 3.2	12.1 ± 3.1	50.0	114.0 ± 3.9	61.2	75.1
PG	12.9 ± 5.2	17.5 ± 6.3	50.0	91.0 ± 3.4	67.3	77.7

* average of three validation levels with standard deviation (± SD).

**Table 3:** Results of muscle, liver, lung, and kidney samples collected during depletion study. Tissues were collected after a single oral administration of acetaminophen at 10 mg/kg in geese. Obtained values are presented as mean value (*n* = 8) ± standard deviation.

Matrix	APAP µg/kg	PS µg/kg	PG µg/kg
Muscle	174 ± 24	136 ± 30	106 ± 21
Liver	918 ± 73	< LOQ	765 ± 30
Lung	494 ± 71	966 ± 86	1120 ± 97
Kidneys	< LOQ	< LOQ	257 ± 86

## Data Availability

The data presented in this study are available on request from the corresponding author.
